# Psychosocial Crowding Stress-Induced Changes in Synaptic Transmission and Glutamate Receptor Expression in the Rat Frontal Cortex

**DOI:** 10.3390/biom11020294

**Published:** 2021-02-16

**Authors:** Agnieszka Zelek-Molik, Bartosz Bobula, Anna Gądek-Michalska, Katarzyna Chorązka, Adam Bielawski, Justyna Kuśmierczyk, Marcin Siwiec, Michał Wilczkowski, Grzegorz Hess, Irena Nalepa

**Affiliations:** 1 Department of Brain Biochemistry, Maj Institute of Pharmacology, Polish Academy of Sciences, Smętna 12, 31-343 Krakow, Poland; wiatrow@if-pan.krakow.pl (K.C.); bielaw@if-pan.krakow.pl (A.B.); justyna.kusmierczyk@awf.krakow.pl (J.K.); wilczkow@if-pan.krakow.pl (M.W.); nalepa@if-pan.krakow.pl (I.N.); 2 Department of Physiology, Maj Institute of Pharmacology, Polish Academy of Sciences, Smętna 12, 31-343 Krakow, Poland; bobula@if-pan.krakow.pl (B.B.); gadek@if-pan.krakow.pl (A.G.-M.); siwiec@if-pan.krakow.pl (M.S.); hess@if-pan.krakow.pl (G.H.)

**Keywords:** glutamate receptors, glutamate transporters, long-term potentiation, paired-pulse ratio, crowding stress, frontal cortex

## Abstract

This study demonstrates how exposure to psychosocial crowding stress (CS) for 3, 7, and 14 days affects glutamate synapse functioning and signal transduction in the frontal cortex (FC) of rats. CS effects on synaptic activity were evaluated in FC slices of the primary motor cortex (M1) by measuring field potential (FP) amplitude, paired-pulse ratio (PPR), and long-term potentiation (LTP). Protein expression of GluA1, GluN2B mGluR1a/5, VGLUT1, and VGLUT2 was assessed in FC by western blot. The body’s response to CS was evaluated by measuring body weight and the plasma level of plasma corticosterone (CORT), adrenocorticotropic hormone (ACTH), and interleukin 1 beta (IL1B). CS 3 14d increased FP and attenuated LTP in M1, while PPR was augmented in CS 14d. The expression of GluA1, GluN2B, and mGluR1a/5 was up-regulated in CS 3d and downregulated in CS 14d. VGLUTs expression tended to increase in CS 7d. The failure to blunt the effects of chronic CS on FP and LTP in M1 suggests the impairment of habituation mechanisms by psychosocial stressors. PPR augmented by chronic CS with increased VGLUTs level in the CS 7d indicates that prolonged CS exposure changed presynaptic signaling within the FC. The CS bidirectional profile of changes in glutamate receptors’ expression seems to be a common mechanism evoked by stress in the FC.

## 1. Introduction

Data showing effects of social stress in animal models are scarce and limited to models that are based on strong social interactions and related with behaviors like isolation, aggression, or subordination [1]. The psychosocial model of crowding stress (CS) is connected with the proximity to companions and competition for access to drinking water or food rather than the expression of antagonistic behaviors. For this reason, the CS model is similar to common social stressors in humans that may lead to stress-related pathology. Moreover, data obtained from the CS model suggest that animals exposed chronically to CS do not develop habituation or homeostatic adaptation to crowd exposure—which is a typical response to physical stressors, observed on the level of endocrine signaling, synaptic transmission, and intracellular signal transduction [2,3,4].

The frontal cortex (FC) is a brain region responsible for executive functions and is known to be impaired in stress-related disorders [5]. It was shown that increased FC activity is necessary for the reduction of magnitude and the duration of the stress response [6,7] in human everyday existence. On the other hand, reduced FC activity (hypofrontality) is considered to underlie stress-related disorders, like depression [8], post-traumatic stress disorder [9], addiction [10], or schizophrenia [11]. Similarly, biphasic effects of stress on FC glutamatergic synaptic transmission and neuronal morphology were documented using preclinical animal models employing mostly stressors that are physical in nature (reviewed in [12]). The function of glutamatergic signaling systems highly depends on the density and activity of glutamate receptors, namely the ionotropic α-amino-3-hydroxy-5-methyl-4-isoxazolepropionic acid (AMPA) receptors, N-methyl-d-aspartate (NMDA) receptors, as well as metabotropic glutamate receptors (mGluR). Functional AMPA receptors are tetramers with GluA1/2 that are the predominant subtypes in many neurons, and the excitatory postsynaptic potential depends mainly on GluA1 subunits [13]. Similarly, NMDA receptorss are most commonly composed of GluN1/2 heterodimers [14]. GluN1 is obligatory in the heterodimer, while the expression of the four different subtypes of GluN2 varies spatially and developmentally [15]. GluN2A and GluN2B are the most prominent GluN2 subunits present in the adult rodent forebrain [15]. Pharmacological, genetic, and lesion studies in animals point to the importance of GluN2B-containing NMDA receptors, especially those expressed in the FC, for stress-related cellular adaptation [16,17]. Pharmacological data have also indicated the crucial role of metabotropic receptors in the pathophysiology of stress-related disorders [18]. Group I mGluRs—mGluR1 and mGluR5—which transmit intracellular signals via Gq/11 heterotrimeric G proteins, seem to be of special importance. Group I mGluRs are localized postsynaptically on neurons in the perisynaptic zone surrounding the ionotropic receptors. The functional form of these receptors exists as a hetero- or homodimer [19], that participates in synaptic long-term plasticity [20,21].

Our previous data concerning the CS model revealed that stimulating the stress axis with CRH injection (1ug/kg) augmented serum levels of CORT in CS 3d and CS 7d groups in a similar manner, while adrenocorticotropic hormone (ACTH) levels were higher in CS 7d than in CS 3d [22]. There are currently no electrophysiological studies showing CS effects on FC synaptic transmission. Therefore, we measured field potentials evoked by a single or paired stimuli in FC ex vivo slices obtained from animals exposed to CS for 3, 7, and 14 days to test the assumption that animals cannot adapt to prolonged CS exposure. Moreover, we investigated the effects of CS on the magnitude of long-term synaptic potentiation in FC slices and assessed protein expression levels for selected subunits of glutamate receptors: GluA1, GluN2B, mGluR5/1a, as well as vesicular glutamate transporters: VGLUT1 and VGLUT2. The stress reaction during the CS procedure was monitored by the analysis of body-weight gain, and hypothalamic-pituitary-adrenal axis (HPA) activation was assessed by measuring plasma corticosterone (CORT), ACTH, and interleukin 1 beta (IL1B) levels. It is not clear whether IL-1B secreted peripherally is engaged in psychosocial stress pathology [23,24]. Nevertheless, the presence of this pro-inflammatory cytokine in the FC was shown to be implicated in stress-induced changes in the excitatory transmission in the rat frontal cortex [25]. Therefore, we also assessed the serum level of IL-1B in CS groups.

## 2. Materials and Methods

### 2.1. Animals

Taking into account that the animals in our study served as a model of stress-related pathologies and male rats are more susceptible to stress associated with housing in groups than females [26], we conducted our study using male Wistar rats (Charles River Laboratories, Sulzfeld, Germany). All rats from two cohorts were used to obtain current data, and, at the beginning of the experiments, the animals were 6 weeks old, weighing 190–220 g. The animals were housed with unlimited access to commercial food and tap water in standard rat cages (Uno Housing, Zevenaar, Netherlands). The following standard laboratory conditions were maintained in the animal room: an artificial 12-h light/dark cycle (lights on from 7 a.m. to 7 p.m.) and at constant temperature 22 ± 2 °C. Before the onset of the experiments, the animals were allowed 1 week of habituation period. All procedures were approved by the Local Ethical Commission for Animal Experiments at Maj Institute of Pharmacology, at the Polish Academy of Sciences, in Krakow (Permit No. 250/2018, date 01 Aug 2018 and 130/2019, date 30 May 2019), and fulfilled the requirements of the EU Directive 2010/63/EU on the protection of animals used for scientific purposes.

### 2.2. Stress Procedures

Rats were randomly assigned to control and experimental stress groups. Control animals were kept in cages under standard conditions (312 cm^2^ per rat) and were not subjected to any stress. The rats from CS groups were kept in cages under overcrowded conditions (70 cm^2^ per rat) for 3, 7, or 14 consecutive days [27]. To check rats’ well-being and assess the stress reaction, animals’ body weight was monitored every second day. For electrophysiology, two separate cohorts of rats were used. In the first cohort (*n* = 9) the effect of CS lasting for 3 days was investigated, whereas the second cohort (*n* = 25) was used to assess the effects of CS lasting for 7 and 14 days.

### 2.3. Tissue Processing

After the stress procedure, all rats were transferred to separate cages for 1 h and decapitated after that period. Trunk blood from the decapitation wound was collected between 12 p.m. and 2 p.m. into EDTA-coated tubes (Profilab, Warsaw, Poland) for stress hormone measurement. From three control and four CS 3d rats adrenals, the spleen and thymus were removed and weighed for further calculation of their weight (in mg) relative to body mass (in g) to obtain the adrenal gland, spleen, and thymus index—considered as physiological stress markers. Adrenals were then immersed in 4% paraformaldehyde necessary for further paraffin embedding and adrenal gland morphometric analysis. The brains were rapidly removed from the skulls. The frontal cortex (FC, rostral from Bregma 2.7 mm without the olfactory bulb) destined for immunoblotting was excised on an ice-cold glass plate, immediately frozen on dry ice, and stored at −70 °C until assayed. The brains destined for electrophysiological studies were immersed in an ice-cold artificial cerebrospinal fluid (ACSF) with the following composition (in mM): NaCl (130), KCl (5), CaCl_2_ (2.5), MgSO_4_ (1.3), KH_2_PO_4_ (1.25), NaHCO_3_ (26), and d-glucose (10), bubbled with a mixture of 95% O_2_ and 5% CO_2_. Frontal cortical slices (420 μm thick) were cut in the coronal plane using a vibrating microtome (Leica, Wetzlar Germany). Slices were stored at 32 ± 0.5 °C and then placed in an interface recording chamber superfused (2.5 mL/min) with a modified ACSF (temperature: 32 ± 0.5 °C) containing (in mM): NaCl (132), KCl (2), CaCl_2_ (2.5), MgSO_4_ (1.3), KH_2_PO_4_ (1.25), NaHCO_3_ (26), and d-glucose (10).

### 2.4. ELISA Analysis of CORT, ACTH, and IL1B Levels

Plasma from blood samples was isolated according to a previously described protocol [28]. Briefly, blood samples were centrifuged at 3000× *g* for 15 min at 4 °C, and then the plasma was transferred to new 1.5 mL collection tubes and stored at −20 °C. CORT, ACTH, and IL 1B concentrations were determined with an enzyme-linked immunosorbent assay (ELISA) method using commercially available Rat Corticosterone ELISA, Rat Adrenocorticotropic Hormone ELISA, and Rat IL1B ELISA kits (Bioassay Technology Laboratory, Shanghai, China). The immunoenzymatic reaction was prepared and developed according to the manufacturer’s instructions. Absorbance was determined at 450 nm using a plate reader (Synergy MX, Biotek, Winooski, VT, USA) and hormone concentrations in duplicates for each sample were determined from standard curves fitted with four parameter logistic equations in the Graph Pad Prism 5.0 (GraphPad Software, San Diego, CA, USA)

### 2.5. Morphometric Analysis of the Adrenal Glands

The adrenal processing was described elsewhere [29]. Briefly, the tissues were fixed in 4% paraformaldehyde overnight, embedded in paraffin, and sectioned on a rotary microtome at a nominal thickness of 7 µm. Every 21st section spanning the entire adrenal (approximately 12–14 sections per adrenal) was stained with hematoxylin and eosin and subjected to morphometric analysis using the ImageJ and NIS Elements BR 3.0 software coupled to a CCD (Charge-Coupled Device) camera mounted on a Nikon Eclipse 50i microscope (Nikon Instruments Inc., Tokyo, Japan). The cross-sectional area of the cortex and medulla was measured in each investigated section. The volume of the cortex and medulla in relation to the body mass for each animal was calculated by multiplying the measured area by the thickness of the section and the fraction of the total volume that it represented (total volume of 21 sections as every 21st section was stained) and dividing the result by the animal’s weight on the last day of the experiment.

### 2.6. Field Potential, Paired-Pulse Stimulation Recordings, Long-Term Potentiation (LTP) Induction, and Data Analysis

For FP recording, a bipolar stimulating electrode (FHC) was placed approx. 2 mm lateral to the midline and approx. 1.5 mm below the pial surface (in layer V). Stimuli (duration: 0.2 ms) were applied at 0.033 Hz using a constant-current stimulus isolation unit (WPI). FPs were recorded using glass micropipettes filled with ACSF (1–2 MΩ), which were placed approx. 0.3 mm below the cortical surface (in layer II/III). FPs were amplified (Axoprobe 1A, Axon Instruments, Burlingame, CA, USA), A/D converted at 10 kHz, and stored using the Micro1401 interface and Signal 4 software (CED, Cambridge, UK).

The stimulus–response curves obtained for each slice were fit with the Boltzmann equation: Vi = Vmax/(1 + exp((u − uh)/−S), with Vmax being the maximum FP amplitude; u—stimulation intensity; uh—stimulation intensity evoking the FP of half-maximum amplitude; S—factor proportional to the slope of the curve. The threshold stimulation was determined as the stimulus intensity necessary to evoke the FP of approximately 0.1 mV in amplitude. The results are expressed as the means ± SEM. The student’s *t*-test, a one-way ANOVA followed by the Tukey’s multiple comparisons test, or a post-hoc Tamhane’s T2 multiple comparisons test following a Welch’s ANOVA test were used to evaluate differences between groups. For the measurement of the short-term plasticity, the paired-pulse stimulation protocol was used (duration—0.2 ms, inter-stimulus interval ISI—50ms). Stimulation intensity was adjusted to evoke a response of 30% of the maximum FP amplitude. The paired-pulse ratio (PPR) was expressed as the ratio of the amplitude of the second FP to the amplitude of the first FP.

For the induction of LTP, theta burst stimulation (TBS) was used. The stimulation intensity was adjusted to evoke a response of 30% of the maximum FP amplitude. TBS consisted of 10 trains of stimuli at 5 Hz, each composed of five pulses at 100 Hz, repeated five times every 15 s. During TBS the pulse duration was increased to 0.3 ms. LTP magnitude was determined as an average increase in the amplitude of the FPs recorded between 45 and 60 min after TBS, relative to the baseline.

### 2.7. Immunoblotting Data Generation and Analysis

Total protein was extracted with Radioimmunoprecipitation assay (RIPA) buffer (MilliporeSigma, Burlington, MA, USA) according to the Gadek–Michalska protocol [30]. Equal amounts of protein extracts were diluted with a loading buffer containing an inclusion body solubilization buffer (G-Biosciences, Saint Louis, MO, USA) and a reducing agent, 1% 2-mercaptoethanol. The samples in the loading buffer were denatured at 45 °C for 30 min to prevent the dissociation of mGluR dimers and the aggregation of hydrophobic transporter proteins [31]. Next, a standard western blot procedure was conducted as previously described [32]. Briefly, denatured samples were run on SDS-PAGE gels, then transferred to nitrocelulose membranes. Membranes were then blocked with 5% nonfat dry milk in Tris-buffered saline with 0.1% Tween-20 (TBST; pH = 7.6) for 1 h at room temperature and incubated with specific primary antibodies. All studied glutamatergic receptors were assessed on the same membranes. After staining with Ponceau S, the membranes were cut horizontally on the 150 kDa level and the proteins—mGluR I dimer (250 kDa), *p*- and total GluN2B (180 kDa)—were assessed on the upper piece, while GluA1 (106kDa) was assessed on the bottom part. The expression of glutamate transporters was assessed on separate gels. After overnight incubation at 4 °C with primary antibodies and three washes with blocking solution, the membranes were incubated with their appropriate secondary antibodies for 1 h at room temperature, followed by three washes with TBST. Antibody binding was detected using an enhanced chemiluminescence kit (ECL Plus 32106, Thermo Fisher Scientific, Inc., Waltham, MA, USA, USA). Equal loading proteins were further confirmed by probing with anti-calnexin antiserum (1:5000; ADI-SPA-865-F, Enzo Life Sciences, Farmingdale, NY, USA) or anti-β-actin antiserum (1:5000; A5441, MilliporeSigma, MilliporeSigma, Burlington, MA, USA). The following antibodies were used in the experiment: p(Y1472) GluN2B (1:1000, M2442, MilliporeSigma, MilliporeSigma, Burlington, MA, USA), GluN2B (1:1000, 610416, BD Biosciences, San Jose, CA, USA), mGluR5/1a (1:2000, 2032-mGluR5/1a, Phosphosolution, Aurora, CO, USA), GluA1 (1:2000, ab31232 abcam, Great Britain), VGLUT1 (1:1000, MAB5502, MilliporeSigma, MilliporeSigma, Burlington, MA, USA), and VGLUT2 (1:1000, D7D2H, Cell Signaling Technology, Danvers, MA, USA). All western blot analyses were performed at least twice to confirm the results. The chemiluminescence of specific signals was visualized with the Multi-Application Gel Imaging System, and the immunoreactive bands were quantified by an image analyzer (MultiGauge V3.0, Fujifilm, Tokyo, Japan).

All values represent the percentage of controls and are expressed as the means ± standard error of the mean (SEM), where group size amounted to *n* = 4–9 rats. Statistical analyses were performed using the Statistica 10 (Round Rock, TX, USA). Data were assessed by a one-way analysis of variance (ANOVA) followed by a post-hoc test when appropriate; *p* < 0.05 was considered as a significant effect.

## 3. Results

### 3.1. Rat Body Response to Crowding Stress Exposure

#### 3.1.1. Body Weight

The initial mean body weight ± SEM of the animal groups analyzed in the experiment was as follows: control for CS3d—185 ± 2.99 g, *n* = 12; CS 3d—192 ± 2.99 g, *n* = 12; control for CS 7d and CS 14d—210 ± 2.03 g, *n* = 7; CS 7d—216 ± 2.34 g, *n* = 24; CS 14d—212 ± 1.80 g, *n* = 24. The unpaired *t*-test revealed that the body weight gain was significantly reduced in CS 3d (*t*(34) = 12.97; *p* < 0.0001), CS 7d (*t*(27) = 6.86; *p* < 0.0001), and CS 14d (*t*(29) = 5.54; *p* < 0.0001) in comparison to control animals.

#### 3.1.2. Plasma Stress Hormone Levels

**Figure 1:**
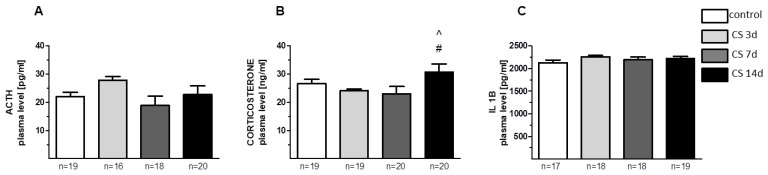
The concentration of adrenocorticotropic hormone (ACTH) (**A**), plasma corticosterone (CORT) (**B**), and interleukin 1 beta (IL1B) (**C**) in plasma isolated from trunk blood of rats that underwent crowding stress (CS) for 3, 7, or 14 days. Bars represent ACTH, CORT, and IL1B concentrations 1 h after the transfer of rats into separate cages and refer to the levels assessed between 1:00 am and 2:00 pm. # *p* < 0.05 vs. CS 3d (LSD test); ^ *p* < 0.05 vs. CS 7d (LSD test). *n* = 16–20/group, as indicated in the graphs.

Control concentrations of stress hormones in blood plasma amounted to 22.05 ± 1.53 pg/mL for ACTH, 26.5 ± 1.59 ng/mL for corticosterone, and 2128.38 ± 1.53 pg/mL for IL1B. A slight increase in the ACTH level was observed only in the CS 3d group (by 24% vs. control) (Figure 1A), but the effect was statistically insignificant [F(3, 69) = 1.89; *p* = 0.14]. A one-way ANOVA for corticosterone concentration among treatment groups revealed an effect on the border of statistical significance [F(3, 74) = 2.62; *p* = 0.057]. A Fisher LSD test showed a significantly increased corticosterone concentration in CS 14d in comparison to the corticosterone levels observed in rats after a shorter exposure to the same stress (by 28% vs. CS 3d and 33% vs. CS 7d (*p* < 0.05)) (Figure 1B). CS did not affect the level of IL1B (Figure 1C).

#### 3.1.3. Adrenal Morphometry, Thymus, and Spleen Weight after 3 Days of Crowding Stress

**Figure 2:**
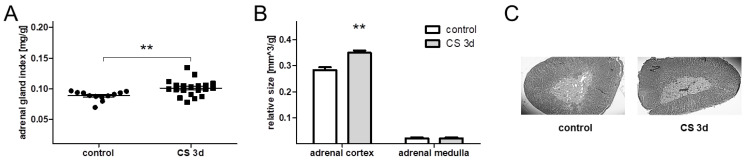
Three-day crowding stress affected adrenal gland morphometry. (**A**) stress effect on the adrenal gland index measured as the ratio of adrenal mass to body weight (*n* = 10); (**B**) stress-evoked increase of the adrenal cortex volume (control: *n* = 3; CS 3d: *n* = 4); (**C**) representative sections of the adrenal gland from control and stressed rats stained with hematoxylin and eosin. ** *p* < 0.01 (unpaired *t*-test).

In order to determine whether CS is stressful after short-term exposure we performed adrenal morphometry and weighed the other stress-sensitive organs—the thymus and spleen in the CS 3d group—and compared the results to those of the control group. The animals subjected to CS 3d had an increased adrenal gland index, calculated as the ratio of the adrenal weight measured in [mg] to body mass measured in [g]: 0.089 ± 0.002 mg/g for controls and 0.101 ± 0.003 mg/g for CS 3d (*t*(30) = 2.95; *p* < 0.006) (Figure 2A). Furthermore, the volume of the adrenal cortex was increased in the CS 3d group vs. control rats. The relative size in [mm^3^/g] was 0.28 ± 0.01 for controls and 0.35 ± 0.009 for the CS 3d group (*t*(5) = 4.45; *p* < 0.01). There were no effects on the relative size of the adrenal medulla (*t*(5) = 0.09; *p* > 0.9) (Figure 2B,C).

Three-day crowding stress also increased the thymus and spleen indexes. The thymus index in the CS 3d group was 2.37 ± 0.12 mg/g (*n* = 12), while in controls it amounted to 2.69 ± 0.10 mg/g (*n* = 20). This effect was on the border of statistical significance (*t*(30) = 2.02; *p* = 0.05). The spleen index in the CS 3d group reached 3.33 ± 0.09 (*n* = 12) and for controls 3.64 ± 0.07 (*n* = 20) (*t*(30) = 2.58; *p* < 0.05).

### 3.2. The Effects of Different Durations of Crowding Stress on Field Potentials

**Figure 3:**
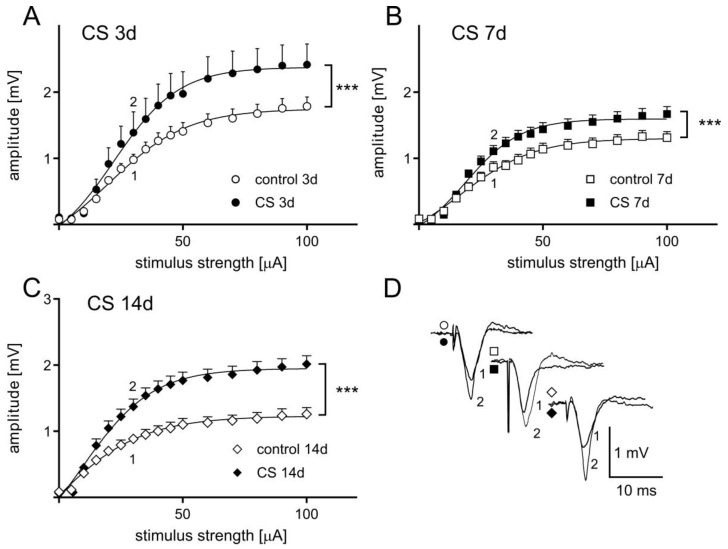
The influence of crowding stress on basal synaptic transmission in the frontal cortex (FC) of rats. The relationship between the stimulus strength and the field potential (FP) amplitude recorded in FC slices from (**A**) control (*n* = 11) and CS 3d (*n* = 8), (**B**) control (*n* = 19) and CS 7d (*n* = 19), (**C**) control (*n* = 19) and CS 14d (*n* = 18). FP traces in (**D**) show a superposition of averaged FPs recorded in representative experiments at the points indicated by numbers. Symbols (circles, squares, and diamonds) correspond to appropriate plots (A–C). Data are presented as means ± SEM and fitted with Boltzmann functions, whereas statistical comparisons are made for the maximum parameter taken from the Boltzmann fit. *** *p* < 0.0001 vs. control (post-hoc Sidak’s test).

Analyses of FPs recorded in slices obtained from rats undergoing crowding stress for 3, 7, or 14 days (Figure 3) revealed a marked increase in the relation between FP amplitude and stimulus intensity (input-output curve) compared to slices obtained from control rats. A two-way ANOVA for FP amplitudes, with the length of stress and the control/stress groups as independent factors, revealed a significant interaction between factors (F(2, 71) = 25.13; *p* < 0.0001). A post-hoc Sidak’s multiple comparisons test revealed that each of the three CS paradigms used had a significant effect on the maximum FP amplitude (3-day CS: *t* = 12.07, *df* = 71.00, *p* < 0.0001; 7-day CS: *t* = 4.367, *df* = 71.00, *p* = 0.0001; 14-day CS: *t* = 10.43, *df* = 71.00, *p* < 0.0001).

### 3.3. The Effects of Different Time Exposure to Crowding Stress on Paired-Pulse Ratio

**Figure 4:**
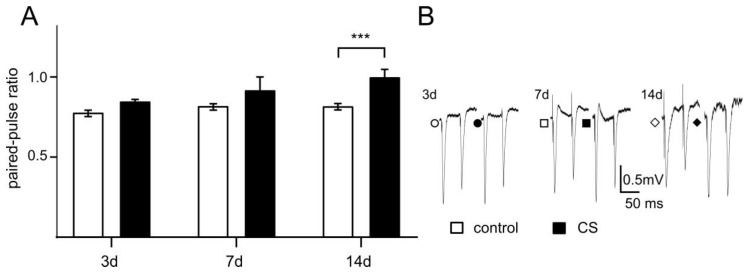
The influence of CS on paired-pulse ratio (PPR) in recordings from FC slices. **A**—Comparison of PPR in CS 3d (*n* = 8) vs. control (*n* = 11); CS 7d (*n* = 19) vs. control (*n* = 19) and CS 14d (*n* = 19) vs. control (*n* = 9), respectively. **B**—Examples of individual FPs evoked by paired stimuli in slices from control (open shapes) and after CS stress (filled shapes). Shapes of symbols corresponding to appropriate animal groups (circles–3 days, squares–7 days, and diamonds–14 days of treatment). *** *p* < 0.0001 vs. control (post-hoc Sidak’s test).

A two-way ANOVA revealed no significant interaction between stress duration and stress-induced changes in PPR (F(2, 71) = 1.518; *p* = 0.2261). However, the main effect of stress on PPR was significant (F(1, 71) = 19.32; *p* < 0.0001, Figure 4). A post-hoc Sidak’s multiple comparisons test revealed significant differences in the PPR between the CS 14d and control groups (*t* = 4.01, *df* = 71.00, *p* = 0.0004). In the other stress groups, the differences were not significant (CS 3d: *t* = 1.362, *df* = 71.00, *p* = 0.4433; CS 7d: *t* = 2.428, *df* = 71, *p* = 0.0522) (Figure 4).

### 3.4. The Effects of Different Durations of Crowding Stress on Long-Term Potentiation

**Figure 5:**
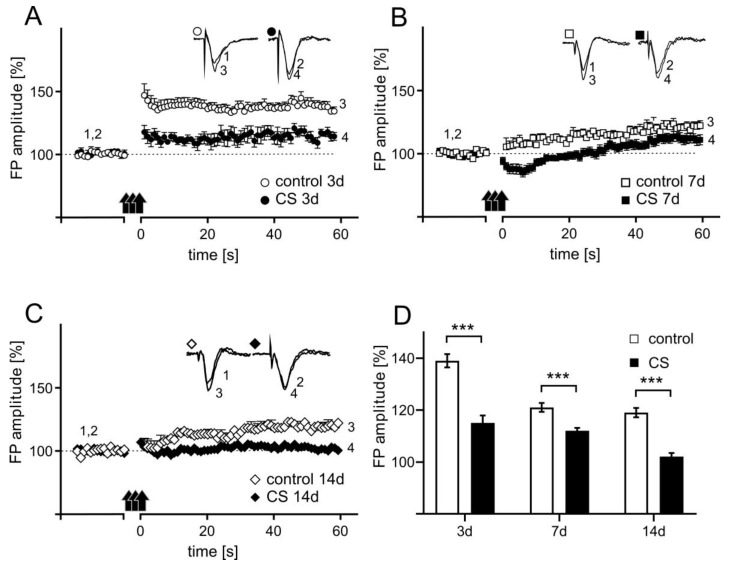
Crowding stress-evoked decrease in LTP magnitude in the rat FC. Plots of the time course of FP amplitude (± SEM) before and after LTP induction showing the influence of CS on LTP after 3 days of CS (**A**) and after 7 (**B**) or 14 days of CS (**C**). The data are presented as group means ± SEM for controls (non-filled shapes) and different stress groups: 3 days (filled circles, *n* = 8), 7 days (filled squares, *n* = 19), and 14 days (filled diamonds, *n* = 18). Insets in A, B, and C show the superposition of averaged FPs recorded in representative experiments at the times indicated by numbers. Black arrows in A, B, and C denote the TBS. (**D**) Comparison of the CS-evoked changes LTP magnitude. Data calculated as a percentage of changes relative to baseline control values (100% indicated as a dashed line). *** *p* < 0.0001.

In slices prepared from rats subjected to CS for 3 days the mean FP amplitude was measured between 45 and 60 min after TBS reached 115 ± 2.9% of the baseline, a value significantly lower than the LTP induced in control slices (137 ± 3.9% of baseline; Figure 5A). In slices prepared from animals that belonging to control groups for CS 7d and CS 14d experimental groups the magnitude of LTP was smaller (121 ± 1.7% of baseline; Figure 5B and 119 ± 1.8% of baseline; Figure 5C, respectively) in comparison to controls for CS 3d group. Nevertheless, in slices obtained from rats subjected to CS for 7 and 14 days LTP was further attenuated (112 ± 1.1% of baseline; Figure 5B and 102 ± 1.4% of baseline; Figure 5C, respectively).

A two-way ANOVA for LTP magnitude with the length of stress and the control/stress groups as independent factors revealed a significant interaction between factors (F(2, 71) = 25.13; *p* < 0.0001, Figure 5). A post-hoc Sidak’s multiple comparisons test revealed that each of the three CS paradigms used had a significant impact on LTP magnitude (CS 3d: *t* = 8.191, *df* = 71.00, *p* < 0.0001; CS 7d: *t* = 3.871, *df* = 71.00, *p* = 0.0007; CS 14d: *t* = 6.603, *df* = 71.00, *p* < 0.0001).

### 3.5. The Effects of Different Durations of Crowding Stress on GluA1, GluN2B, and mGluR5/1a Expression in the Rat FC

#### 3.5.1. GluA1 Protein

**Figure 6:**
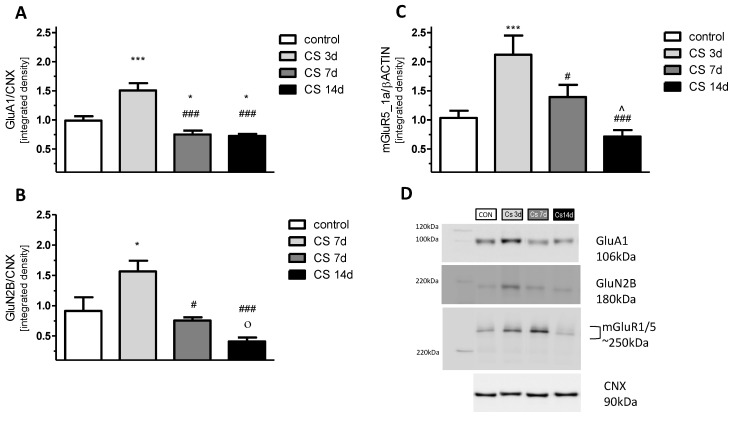
The influence of crowding stress (CS) and its duration on the expression of selected glutamate receptors in the rat FC. Expression of the GluA1 subunit of the α-amino-3-hydroxy-5-methyl-4-isoxazolepropionic acid (AMPA) receptor (**A**), GluN2B subunit of the n-methyl-d-aspartate (NMDA) receptor (**B**), metabotropic receptor dimer GluR5/1a (**C**). (**D**) Representative immunoblots that illustrate GluA1, GluN2B, and mGluR5/1a expression levels in the FC. Data were calculated as percentages of controls and are expressed as means ± SEM (CON: *N* = 9; CS 3d: *N* = 5; CS 7d: *N* = 5; CS 14d: *N* = 5). *** *p* < 0.001; * *p* ≤ 0.05; ○ 1> *p* >0.05 vs. control (LSD test); ### *p* < 0.001; # *p* < 0.05 vs. SC 3d (LSD test); ^ *p* < 0.05 vs. CS 7d (LSD test).

**Figure 7:**
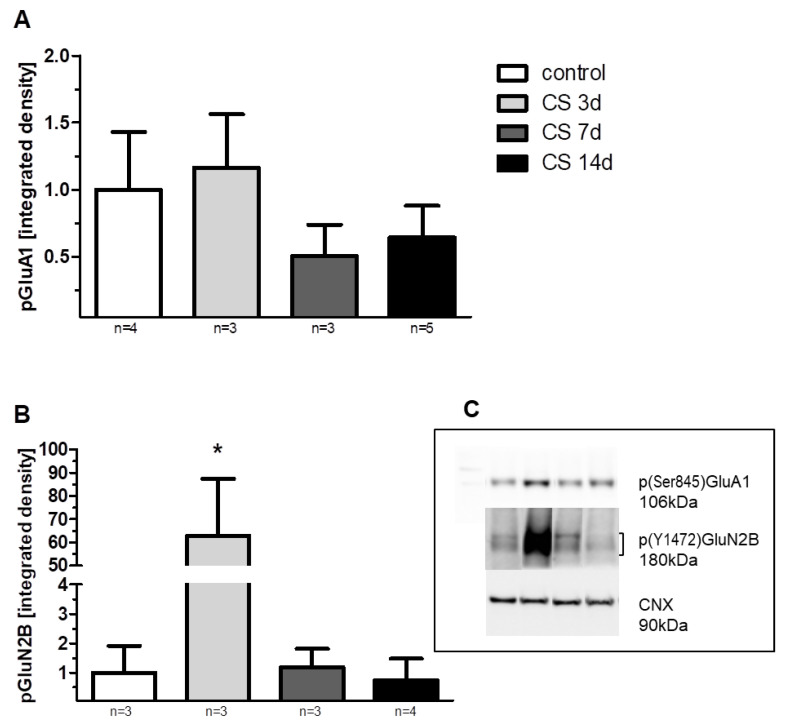
The influence of crowding stress (CS) and its duration on the phosphorylation level of the GluA1 subunit of the AMPA receptor (**A**), GluN2B subunit of the NMDA receptor (**B**). (**C**) Representative immunoblots that illustrate GluA1 and GluN2B phosphorylation levels in the FC of all studied groups. Bands’ order from the left: molecular weight marker; control; CS 3d; CS 7d; CS 14d. Data were calculated as percentages of controls and are expressed as means ± SEM. A one-way ANOVA revealed that the phosphorylation of GluN2B at tyrosine(Y)1472 was significantly elevated in the CS 3d group vs. the other groups [F(3, 9) = 7.31; * *p* < 0.05].

A one-way ANOVA for changes in GluA1 expression levels between different stress durations revealed significant differences among the analyzed groups [F(3, 20) = 15.5; *p* < 0.01]. A post-hoc Fisher LSD test pointed out a significantly increased GluA1 expression (by 50%) vs. control in CS 3 d (*p* < 0.01). After the initial induction of GluA1 expression observed in the CS 3d group, the longer exposure to crowding stress, 7 or 14 days, decreased GluA1 expression by 20% vs. control. A post-hoc analysis showed that GluA1 levels in both the CS 7 d and CS 14d groups were significantly lower vs. the CS 3 d group (*p* < 0.001, LSD test) and were also decreased on the border of statistical significance vs. the control group (0.1 > *p* > 0.05, LSD test) (Figure 6A,D). As the experiment involved measuring protein expression isolated from all cellular fractions (protein extraction by RIPA), to estimate whether the observed changes in GluA1 expression were related to its presence in the membrane, we also checked the phosphorylation level of p(Ser845)GluA1. The profile of CS-evoked changes in the phosphorylation of GluA1 at Serine-845 was similar to the CS-evoked alterations in GluA1 expression. There were no statistically significant differences in GluA1 phosphorylation levels among groups (Figure 7A).

#### 3.5.2. GluN2B Protein

A one-way ANOVA revealed significant differences among the analyzed groups [F(3, 20) = 5.3; *p* < 0.01]. A post-hoc Fisher LSD test indicated significantly increased GluN2B expression (by 71%) vs. control in the CS 3d group (*p* < 0.05). The observed increase of the expression of GluN2B subunits in the CS 3d group was gradually decreased in proportion to the length of CS exposure and was 17% and 55% lower than control levels in the CS 7d and CS 14d groups, respectively. For both the CS 7d and CS 14d groups, GluN2B expression was significantly decreased vs. the CS 3d group (*p* < 0.05, *p* < 0.01 respectively, LSD test) (Figure 6B,D). Additionally, there was a decrease in the GluN2B expression in the CS 14d group on the border of statistical significance vs. the control group (0.1 > *p* > 0.05, LSD test). We observed a similar increase of phosphorylation levels of p(Y1472)GluN2B evoked by CS (increase in CS 3d vs. other groups [F(3, 9) = 7.31; *p* < 0.01] (Figure 7B).

#### 3.5.3. mGluR5/1a Protein Dimer

A one-way ANOVA for mGluR5/1a dimer expression levels revealed significant differences among the analyzed groups [F(3, 20) = 8.8; *p* < 0.01]. A twofold increase in mGluR5/1a expression was observed in the CS 3d vs. the control group (*p* < 0.001, LSD test). Along with the duration of the CS procedure, initially increased levels of mGluR5/1a expression were gradually reversed. mGluR5/1a dimer expression was still 34% higher in the CS 7d group compared with the control group, but its level was significantly lower when compared to the CS 3d group (*p* < 0.01, LSD test). In the CS 14d group, the mGluR5/1a level was 31% lower than in controls, and the observed decrease was statistically significant vs. the CS 3d group (*p* < 0.001, LSD test) and vs. CS 7d (*p* < 0.05, LSD test) (Figure 6C,D).

### 3.6. The Effects of Different Durations of Crowding Stress on the Level of Vesicular Glutamate Transporters VGLUT1 and VGLUT2 in the Rat FC

**Figure 8:**
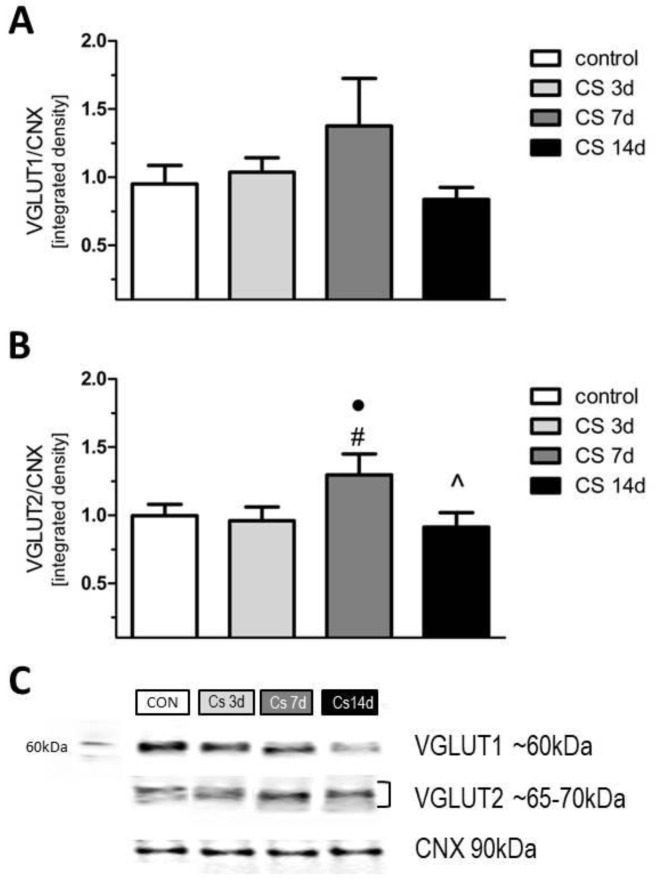
The influence of crowding stress and its duration on the expression of glutamate transporters in the rat FC. Expression of VGLUT1 (**A**), VGLUT2 (**B**). (**C**) Representative immunoblots that illustrate VGLUTs expression in the FC after crowding stress (CS). Data were calculated as percentages of controls and are expressed as means ± SEM (control: *n* = 9; CS 3d: *n* = 5; CS 7d: *n* = 5; CS 14d: *n* = 5) ● 1> *p* > 0.05 vs. control (LSD test); # *p* < 0.05 vs. SC 3days; ^ *p* < 0.05 vs. CS-7days, (LSD test).

Although a one-way ANOVA analysis did not show significant effects for the expression levels of VGLUT1 [F(3, 20) = 1.3; *p* > 0.1] and VGLUT2 [F(3, 20) = 2.1; *p* > 0.1], we noted an increased expression of both VGLUT1 and VGLUT2 (by 30%) in CS 7d vs. control. A post-hoc comparison, which was performed alone, showed that VGLUT2 expression in the CS 7d group was also higher vs. the CS-3d and CS-14d groups (Figure 8).

## 4. Discussion

The present study demonstrates for the first time that CS enhances basal excitatory synaptic transmission and attenuates long-term synaptic plasticity in layer II/III of the primary motor cortex—M1—a dorsal part of the rat FC. On the other hand, the bidirectional effect of psychosocial CS on glutamate receptors’ expression in the FC observed in our study is similar to the effect described in other stress models.

The body weight loss we described in all stressed animals, which had unrestricted access to food and water during the experiment, reflects the profound physiological changes evoked by overcrowding. Literature data describing the mechanism of losing weight in other social stress model (visible burrow system) revealed that persisted weight loss in subordinated rats was accompanied by depressive-like behavior (anhedonia) and metabolic syndrome symptoms [33]. The levels of CORT and ACTH in the CS 3d and CS 7d groups did not show unequivocally the presence of systemic stress reaction. However, in CS 14d rats the CORT level was clearly increased vs. the other groups, indicating the activation of the HPA after prolonged exposure to overcrowding. An increased plasma CORT level after long-term exposure to stress was observed in animals subjected to the chronic mild stress procedure [7] but not in the physical restraint stress model [2,34]. There are several possible reasons why stress hormone levels were not changed in the CS 3d and CS 7d groups. The HPA activity represents a dynamic process, that is rapidly regulated by a feedback mechanism, and the timing of both ACTH and CORT responses depends on the type of stressor [7]. Therefore, the time of our measurement (1 h after the transfer of rats into separate cages) could be too late to detect increases in stress hormone levels. The second possible reason why we did not reveal the rise in stress hormone levels after short-term CS exposure is the presence of other factors inhibiting stress response (e.g., testosterone) [35]. It is noteworthy that in our previous study we recorded augmented levels of stress hormones in the CS 3d and CS 7d groups only when CS rats received an additional boost (CRH ip injection) [36], which was not applied in experiments presented here. Even though short-term stress did not change the plasma level of CORT, in the CS 3d group we showed the increased weight of stress-sensitive organs: the adrenal gland, thymus, and spleen, that is a well-known indicator of experiencing stress [29]. Moreover, the increased volume of the adrenal cortex in the CS 3d group serves as an indirect proof of an increase in CORT production during early exposure to CS. The plasma level of IL-1B was not changed in our study, suggesting that the systemic level of pro-inflammatory cytokines is not implicated in social stress pathology. It should be noted that the results delivered by experiments involving other strains or females could be different than those obtained here, as cytokine effects highly depend on the strain and sex of the animal used in the stress models [37,38].

Basal excitatory synaptic transmission, measured as the input/output curve, was increased in our study in the M1 of stressed rats from the CS 3d, CS 7d, and CS 14d groups. This result is consistent with data obtained in the M1 of rats with experimentally-induced cortisolemia [39] and in adult offspring rats that were prenatally stressed [40]. The FP amplitude in M1 altered by social crowding stress demonstrated here contradicts the effects evoked by repeated physical restraint stress, as an increase in FP amplitude in M1 was noted after short-term stress only [25]. Longer exposure to restraint stress normalized cellular activation in the hypothalamus [2] and FC [4], as evidenced by electrophysiological and immunohistochemical studies. Persistently increased basal synaptic transmission in CS groups could be related to the increased release of glutamate in the FC. Indeed, it was documented that FC is a preferential place for increased glutamate release after stress exposure [41]. Moreover, it has been postulated that the stress-induced elevation of the level of this neurotransmitter is an important factor of mental pathology [42] and riluzol—a known inhibitor of glutamate release—is an effective antidepressant [43].

In animal stress models, increased FP amplitude in M1 was accompanied by the expression of depressive-like behavior [40]. Moreover, it was shown that antidepressant therapies reverse or counteract the stress-evoked increase in synaptic excitability [27,39,44,45]. On the synaptic level, stress increased the frequency of mEPSCs in M1 [39,40], which is related to the enhanced neurotransmitter release. However, in our study, PPR was unchanged in the CS 3d group. It was slightly elevated in the CS 7d group and increased significantly after longer chronic exposure to psychosocial stress—in CS 14d. Changes in PPR are used as a measure of modifications in neurotransmitter release probability [46]. Taken together, the persistently increased amplitude of FP we observed in our study appears to reflect complex, dynamic CS-related changes involving both glutamate release from presynaptic terminals and elevated expression of postsynaptic glutamate receptors. The results obtained here suggest that the mechanisms underlying the effects of CS 3d on basal synaptic transmission are mostly postsynaptic and involve the enhanced expression of the AMPA receptor. At the same time, glutamate release appears unchanged (no change in PPR). Increased basal synaptic transmission is most likely a consequence of the enhanced AMPA receptor activity, because in presence of Mg^2+^ (1.3 mM) in the ACSF, NMDA receptor-mediated currents are blocked [47]. However, the data also show an increase in postsynaptic NMDA and metabotropic glutamate receptor expression. In contrast, the mechanisms underlying the effects of CS 14d on basal synaptic transmission appear to be mostly presynaptic, given the significant change in PPR and increased expression of glutamate transporters accompanied by a decreased expression of postsynaptic glutamate receptors. It is tempting to speculate that the effects on PPR observed after CS 7d result from a decrease of the postsynaptic and increase of the presynaptic contributions. Some available data indicate that the stress-evoked upregulation of presynaptic signaling in the FC is implicated in the mechanism of depression and other stress-related psychiatric disorders. It was shown, among others findings, that prenatal stress upregulated SNAP-25 (Synaptosomal-Associated Protein, 25 kDa)—an element of the presynaptic SNARE (SNAP Receptor proteins) complex in rats [48]. Additionally, maladaptive stress overactivated voltage-dependent calcium channels (N and P/Q types) [43], which are presynaptically located.

The reduction of LTP in M1 presented here is consistent with the effect produced after the short-time exposure of rats to restraint stress (3 days) [25] and with results observed in adult rats stressed prenatally [40]. Moreover, LTP reduction in M1 was detected in rats after chronic treatment with corticosterone [39] in a manner dependent on the intact activity of the glucocorticoid receptor (GR) [49]. The role of stress-evoked LTP modulation is not well understood. Studies involving animal models showed that the induction of LTP in stress-related brain structures is a dynamic process and depends on the activity of different receptors. The exposure of mice to repeated neck restraint stress attenuated LTP in the hippocampus after three stress sessions, and this effect depended on GR activity. After seven sessions, LTP returned to the control level, while a longer exposure to neck restraint stress (for 14 and 21 days) potentiated LTP and was related to mineralocorticoid receptor (MR) activity [47]. In turn, studying saturating levels of LTP and LTD in the rat amygdala showed that early life stress shifted synaptic plasticity in the amygdala cortical inputs toward LTD [50]. Because LA and M1 are directly interconnected [51,52], the stress-evoked changes in synaptic plasticity observed in LA seem to be related to M1 plasticity and described early life stress. Literature data show that stress-evoked LTP reduction, observed in the FC, hippocampus, and amygdala [40,53], has been accompanied by depressive-like behavior and antidepressant treatment normalized LTP level [39,54]. The attenuated possibility of the induction of LTP we observed after CS exposure could resemble the occlusion of LTP occurring as a consequence of learning processes, when enhanced signal transmission and a saturation of the LTP mechanism take place [39]. It was demonstrated that strong synaptic connections in the neocortex do not undergo plasticity, while weak connections tend to be potentiated by presynaptic mechanisms [39]. Taken together, this information suggests that the stress-evoked reduction in LTP noted in our study may be involved in the enhancement of learning processes occurring in the FC and related to increased presynaptic activity.

Our biochemical results revealed that sub-chronic exposure to social stress (CS-3d group) increased the expression of the GluA1 and GluN2B subunits, that was accompanied by a higher amplitude of the FPs of FC neurons than in control animals, as observed in electrophysiology. A similar up-regulation of GluN2A/GluN2B and GluA1 subunits co-occurring with the potentiation of the postsynaptic NMDA and AMPA receptors currents in the FC of rats was accompanied by a facilitation of behavioral performance of tasks after acute exposure to different kinds of stressor [55]. As it is well established that the GluA1 and GluN2B subunits modulate the membrane stability of the ionotropic receptors, their augmented expression correlating with the increased amplitude of FPs in the FC of the CS 3d group could reflect the potentiation of FC glutamatergic transmission due to short-term stress exposure. It should be mentioned that the expression of the glutamate subunits of the ionotropic receptors covered not only the membrane fraction, which is known to be related to synaptic electrophysiological properties, but also other intracellular compartments, where GluA1 and GluN2B are synthesized and processed [56]. However, in our study, CS effects seem to be specific to the membrane AMPA and NMDA receptors containing the GluA1 and GluN2B subunits because CS similarly affected the phosphorylation level of these subunits, which takes place only at the plasma membrane [57]. As it is known that mGluR1a/5s participate in synaptic weakening, the increase in their expression in the CS 3d group suggests the involvement of mGluR signaling in the LTP downregulation we observed. It was documented that mGluR I mediates the so-called depotentiation, that relays on a reduction of transmission at synapses which were previously potentiated [20,21]. The reduced LTP occurring with an increased mGluR5/1a protein level in the FC of rats in the CS 3d group observed in our study may indicate the occurrence of plastic changes manifested by a reduction in synaptic transmission after short-term social CS exposure. The results obtained in the FC of the CS 7d and CS 14d groups were more complex than in the CS 3d group. Increased synaptic activity and impaired LTP were accompanied by a decreased expression of all studied glutamate receptors in the FC in comparison to the control. The alterations in the expression of the glutamate receptors evoked by CS we received are similar to changes triggered in other stress models, where an increase in the glutamate receptors expression evoked by initial, acute stress was followed by downregulation after chronic stress exposure (reviewed by [12]). Results delivered by studies on chronic physical homotypic restraint stress and unpredictable stress models suggest that the loss of glutamate receptors in the FC and the impairments in synaptic excitability result from the increased ubiquitin/proteasome–mediated degradation of the ionotropic receptors [58]. The mechanism responsible for synaptic transmission disturbance in the FC evoked by the chronic social crowding stress described here seems to be different than the one mentioned above. In our studies, the downregulation of GluA1, GluN2B and mGluR1a/5 induced by prolonged CS is accompanied by the augmented amplitude of FP. Our results suggest that chronic CS evoked an increase in FP amplitude as a consequence of augmented presynaptic transmission because the paired-pulse inhibition ratio—commonly regarded as an indicator of the function of presynaptic neurotransmitter release machinery—was increased in the CS 14d group. Moreover, chronic CS upregulated the expression of presynaptic protein VGLUT2 in the FC— which is considered to be a marker of thalamocortical [59] or mesocortical projections [60], indicating that chronic CS is responsible for the neurotransmission increase of thalamic or ventral tegmental area (VTA) glutamatergic inputs.

The limitation of the study presented here is concerned with the fact that the electrophysiological experiments were performed in only one part of the FC (M1), while protein expression was assessed for a larger area of the FC (rostral part from Bregma 2.7 mm without the olfactory bulb). Transcranial magnetic stimulation (TMS) studies in humans focused on motor cortical excitability in psychiatric disorders revealed significant alterations in the functioning of the M1 region in stress-related disorders like depression and PTSD [61]. Additionally, our previous electrophysiological experiments indicating that imipramine reverses impairments in synaptic activity in the FC evoked by chronic corticosterone were performed in M1 [39]. In turn, most biochemical data showing bi-directional effects of physical stress on glutamate receptor expression regard the PFC/FC (reviewed by [12]); therefore, to compare our results from the psychosocial CS model and validate data obtained from other stress models, we analyzed the area of the FC larger than M1. Further experiments should indicate whether and to what extent the immunoblotting results concerning glutamate receptor expression levels performed for the whole FC differ from results performed for the M1 part only.

## 5. Conclusions

Our study provides new information about time-dependent changes in the synaptic properties and expression levels of selected subunits of glutamate receptors as well as vesicular glutamate transporters in the FC of rats undergoing psychosocial stress. We have shown for the first time that, evoked by CS, the enhancement of basal excitatory synaptic transmission and the attenuation of long-term synaptic plasticity are not blunted alongside the prolonged exposure to homotypic stress, suggesting that the procedure of psychosocial stress does not enable the habituation of animals to stress conditions. It proves that the psychosocial CS model could be a promising candidate to study the mechanisms underlying the development of psychosocial stress-related pathologies. Increased postsynaptic glutamate receptor expression levels were evident only after short-term exposure (CS 3d), correlating to the observed synaptic activity in the FC. Longer CS exposure down-regulated the expression levels. The temporarily increased VGLUT expression in the CS 7d group co-occurring with increases in PPR, especially after a more chronic stress exposure (in CS 7d and CS 14d groups), suggests that longer CS exposure utilizes presynaptic mechanisms to propagate stress signaling. Further studies are necessary to understand the detailed cellular mechanisms involved in the fluctuations in protein expression levels observed here.

## Data Availability

The data presented in this study are available on request from the corresponding author.

## Abbreviations

ACSF	artificial cerebrospinal fluid
ACTH	adrenocorticotropic hormone
AMPA	α-amino-3-hydroxy-5-methyl-4-isoxazolepropionic acid
CORT	corticosterone
CS	animal model of psychosocial crowding stress
ELISA	enzyme-linked immunosorbent assay
FC	frontal cortex
FP	field potentials
GR	glucocorticoid receptor
HPA	hypothalamic-pituitary-adrenal axis
Il 1β	interleukin 1 beta
LTP	long term potentiation
NMDA	N-methyl-d-aspartate
PPR	Paired-pulse ratio
